# Automatic Recognition, Segmentation, and Sex Assignment of Nocturnal Asthmatic Coughs and Cough Epochs in Smartphone Audio Recordings: Observational Field Study

**DOI:** 10.2196/18082

**Published:** 2020-07-14

**Authors:** Filipe Barata, Peter Tinschert, Frank Rassouli, Claudia Steurer-Stey, Elgar Fleisch, Milo Alan Puhan, Martin Brutsche, David Kotz, Tobias Kowatsch

**Affiliations:** 1 Center for Digital Health Interventions Department of Management, Technology, and Economics ETH Zurich Zurich Switzerland; 2 Center for Digital Health Interventions Institute of Technology Management University of St. Gallen St. Gallen Switzerland; 3 Lung Center Cantonal Hospital St. Gallen St. Gallen Switzerland; 4 Institute of Epidemiology Biostatistics and Prevention University of Zurich Zurich Switzerland; 5 mediX Group Practice Zurich Switzerland; 6 Department of Computer Science Dartmouth College Hanover, NH United States; 7 Center for Technology and Digital Health Dartmouth College Hanover, NH United States

**Keywords:** asthma, cough recognition, cough segmentation, sex assignment, deep learning, smartphone, mobile phone

## Abstract

**Background:**

Asthma is one of the most prevalent chronic respiratory diseases. Despite increased investment in treatment, little progress has been made in the early recognition and treatment of asthma exacerbations over the last decade. Nocturnal cough monitoring may provide an opportunity to identify patients at risk for imminent exacerbations. Recently developed approaches enable smartphone-based cough monitoring. These approaches, however, have not undergone longitudinal overnight testing nor have they been specifically evaluated in the context of asthma. Also, the problem of distinguishing partner coughs from patient coughs when two or more people are sleeping in the same room using contact-free audio recordings remains unsolved.

**Objective:**

The objective of this study was to evaluate the automatic recognition and segmentation of nocturnal asthmatic coughs and cough epochs in smartphone-based audio recordings that were collected in the field. We also aimed to distinguish partner coughs from patient coughs in contact-free audio recordings by classifying coughs based on sex.

**Methods:**

We used a convolutional neural network model that we had developed in previous work for automated cough recognition. We further used techniques (such as ensemble learning, minibatch balancing, and thresholding) to address the imbalance in the data set. We evaluated the classifier in a classification task and a segmentation task. The cough-recognition classifier served as the basis for the cough-segmentation classifier from continuous audio recordings. We compared automated cough and cough-epoch counts to human-annotated cough and cough-epoch counts. We employed Gaussian mixture models to build a classifier for cough and cough-epoch signals based on sex.

**Results:**

We recorded audio data from 94 adults with asthma (overall: mean 43 years; SD 16 years; female: 54/94, 57%; male 40/94, 43%). Audio data were recorded by each participant in their everyday environment using a smartphone placed next to their bed; recordings were made over a period of 28 nights. Out of 704,697 sounds, we identified 30,304 sounds as coughs. A total of 26,166 coughs occurred without a 2-second pause between coughs, yielding 8238 cough epochs. The ensemble classifier performed well with a Matthews correlation coefficient of 92% in a pure classification task and achieved comparable cough counts to that of human annotators in the segmentation of coughing. The count difference between automated and human-annotated coughs was a mean –0.1 (95% CI –12.11, 11.91) coughs. The count difference between automated and human-annotated cough epochs was a mean 0.24 (95% CI –3.67, 4.15) cough epochs. The Gaussian mixture model cough epoch–based sex classification performed best yielding an accuracy of 83%.

**Conclusions:**

Our study showed longitudinal nocturnal cough and cough-epoch recognition from nightly recorded smartphone-based audio from adults with asthma. The model distinguishes partner cough from patient cough in contact-free recordings by identifying cough and cough-epoch signals that correspond to the sex of the patient. This research represents a step towards enabling passive and scalable cough monitoring for adults with asthma.

## Introduction

Asthma is one of the most prevalent chronic respiratory diseases [1]; it is estimated to afflict 339 million people worldwide [1]. Despite increased investment in treatment, hospital admissions and mortality rates have remained stable over the last decade [2]. This stagnation highlights the lack of scalable and easy-to-manage diagnostic tools for early recognition of exacerbations [3]. Traditional asthma self-management programs include written plans of action that cover how to recognize and respond to exacerbations [4]. These pen-and-paper approaches have shown improved health outcomes such as reduced hospital admissions, better lung function, fewer asthma symptoms, and less use of rescue medication [4-6]. The implementation of these programs, however, is low in clinical practice, with only 27% of adults with asthma receiving an asthma action plan [7]. Also, patient adherence to written action plans is poor and declines over time [8], and due to the reliance on subjective information in action plans, patients may perceive their symptoms poorly, and thus, underestimate the severity of the disease [9].

Disease control (also referred to as asthma control) is defined as the degree to which symptoms are controlled by treatment [10]. Exacerbations of asthma occur gradually over several days to weeks on a background of poor asthma control [11,12]. Although there is consensus on the importance of longitudinal and objective assessment of asthma control in the home environment, few tools enable objective measurement [3]. Current guidelines for the assessment of asthma control recommend the measurement of peak expiratory flow [13]. Changes in peak expiratory flow can occur up to 2 weeks before an exacerbation [14]. This parameter, however, is dependent on effort [3], and only 5% of adults with asthma measure peak expiratory flow regularly [15]; therefore, it has limited utility. Nocturnal cough is a physiologic parameter which has shown promise for use in the assessment of asthma. Cough is a particularly burdensome asthma symptom [16] and was shown to be associated with asthma severity [17] and a worse prognosis [18]. Cough counts per night (ie, the number of coughs produced by one individual per night) have been associated with the level of asthma control [19]. Moreover, lower levels of asthma control have been correlated with poor quality of life [20] and are a predictor for cost-of-illness [21]. Coughing may also provide valuable information to predict the effects of asthma therapy early on [22]. Thus, nocturnal cough monitoring may play an important role in the prevention of exacerbations and in the personalization of treatment.

The monitoring of coughing by quantifying the number of coughs per unit of time has been attempted by researchers since the 1950s [23]. Although cough events may be counted manually from sound and video recordings, this process is extremely laborious. In consequence, many semi and fully automated cough monitoring systems have been in development since the 1950s. Among the more well-known systems are the Hull Automatic Cough Counter [24], the Leicester Cough Monitor [25], and LifeShirt [26]. The Hull Automatic Cough Counter is a software program written in MATLAB with the ability to differentiate cough from noncough sounds using Sony Walkman tape recordings. Leicester Cough Monitor is based on a free-field microphone necklace that records sound continuously onto a digital recorder. Recordings can then be uploaded onto a computer where an automated cough detection algorithm analyzes them. LifeShirt is an ambulatory cardiorespiratory monitoring system with a unidirectional contact microphone. Though these systems lacked scalability and cost-effectiveness, they showed feasibility with respect to automatic cough detection and counting from audio recordings.

Smartphones are now ubiquitous [27] and are equipped with sensors capable of many types of monitoring with clinically valuable accuracy [28]. Their widespread adoption in all age groups enables them to be used for measurements within different population samples [29]. In addition, they can be used to passively monitor the health status of patients without an additional task of activating a monitor. Recent advances have used only a smartphone and its built-in microphone for cough monitoring [30-32].

To develop an automatic cough monitoring system is challenging due to the rare occurrence of coughing in comparison to the occurrence of other sounds. This natural imbalance of cough and noncough sounds poses two problems. First, it demands high specificity from the cough monitoring system to avoid false alarms from other similar and more frequently occurring sounds. Second, existing classification methods tend to perform poorly on minority-class examples when the data set is highly imbalanced [33]. In addition, experts have suggested sensitivities greater than or equal to 90% as necessary for clinical use [34]. An even greater challenge is to collect sufficient data in the intended context and timeframe to allow realistic assessment of the monitoring system, particularly with respect to respiratory conditions. Some cough monitoring systems have been developed based on data collected under lab conditions by recording voluntary coughs [35-37]. While this may be a valid approach to compare the performance of different classifiers, it may not represent the soundscape of a real-use case. No cough monitoring systems exist that have undergone longitudinal overnight testing nor do any exist that have been specifically evaluated in the context of asthma. Also, depending on the intended mode of use, new challenges arise for cough monitoring—distinguishing partner coughs from patient coughs in a room with two or more people using contact-free recordings is a difficult task. To date, there are no standardized methods, and there are no sufficiently validated cough monitors for general use that are commercially available and clinically acceptable [23].

The study was designed to mimic a real-world use case; data were collected from a smartphone placed on the bedside table in the participant's bedroom. The aim was to build a cough classifier and to evaluate its performance on unseen data. Further, we aimed to use the classifier to segment and count cough events over the course of the night. Building upon previous work [32], we adopted a convolutional neural network architecture , which performed best in comparison to that of other machine learning approaches when using voluntary cough data from different smartphone recordings. We altered the learning part of the algorithm by combining three different techniques from literature to combat the high class imbalance encountered in this real-life data set—ensemble learning [38], minibatch balancing [39], and decision thresholding [40].

Though a smartphone-based nocturnal cough monitoring service may enable passive monitoring in theory, its utility for application in practice depends on whether coughs can be correctly assigned to individuals. Prior research has shown that humans are able to determine whether the source of a cough is male or female based on sound alone [41]. In addition, sex-based differences in signal properties have been measured in cough signals [42]. Epidemiological research suggests that less than 10% of the general population identify as homosexual [43]. Assuming that most people either sleep alone or share their bedroom with a partner, for the vast majority of patients, correctly classifying cough by sex-based properties of the cough signal could allow a cough monitoring system to disregard the coughs that are not from the individual of interest. Therefore, in addition to cough detection, this work examines to what extent coughs can be correctly be classified by sex. This research represents a step towards enabling passive scalable monitoring for people with asthma.

## Methods

### Overview

This study involved the collection of smartphone-recorded audio and daily questionnaire data, the definition and quantification of coughs within that data (data annotation and automated cough recognition and segmentation), sex-based classification, and model performance evaluation.

### Data Collection

We used data collected in a multicenter, longitudinal observational study over a 29-day period (28 nights) [41]. On the first and last day, participants underwent medical examination by health professionals at the study centers. At the start of the study, participants were equipped with a smartphone (Samsung Galaxy A3 2017, SM-A320FL) on which Clara—the study’s chat-based app—was installed. This app was a study-specific enhancement of the mobile app in the open-source MobileCoach behavioral intervention platform [44,45]. At night, the app recorded audio data using the smartphone’s microphone. It also delivered daily questionnaires to the patients, asking them (among other things) whether the participant slept alone.

All participant data were collected by the physician (asthma evaluation data) or the nurse (lung function evaluations) in the study centers and were transferred to an electronic format and stored online on the study server. Nightly sensor data were stored locally on the smartphone. Data were backed up to external hard drives and secure online storage once the participant had completed the study and had returned the smartphone.

The study protocol was reviewed and approved by the *Ethikkommission Ostschweiz*, which is responsible for research on humans in Eastern Switzerland (Business Management System for Ethics Committees ID: 2017–01872).

### Cough Definition and Quantification

With respect to cough monitoring systems, the definition of cough depends on the modality used for monitoring [23]. In this study, we aimed to recognize coughs from sound recordings which can be done in several ways [23]. We focused on two methods: (1) counting explosive cough sounds and (2) counting cough epochs (continuous coughing sounds without a 2-second pause [23]). The latter is derived from the first metric by computing the duration between explosive cough sounds.

### Data Annotation

Before annotation, silence was marked by applying a decibel filter by means of the Audacity software to the recordings. The Sound Finder filter marked sounds below –26 dB as silence with the constraint that the minimum duration of silence between sounds was 1 second. These periods marked as silence served as visual aids for the remainder of the annotation process. Human annotators listened to the smartphone recordings and labeled the periods that were not marked as silence as a cough if an explosive cough sound was identified [23,41].

We used two approaches to verify the quality of the labeling. First, we instructed human annotators to label an acoustic event if they were unsure that it was a cough. If annotators were unsure, the event was discarded and was not considered in the analysis. The remainder of acoustic events were classified as noncoughs. Second, the interrater reliability for the annotators was determined using intraclass correlation. A zero-inflated generalized mixed-effects model with a Poisson response was used [46]. Additional details of the annotation method can be found in Multimedia Appendix 1.

### Cough Recognition and Segmentation

#### Data Set Partitioning for Cough Recognition

When developing neural networks, the split into sets for training, validation, and testing of the model is favored over other approaches that involve cross-validation because of the long training phases of the models; however, this comes at the risk of overfitting the model to the specific data set and a lack of generalizability to unseen samples. To mitigate these effects, we split our data into disjunct data sets which contained a different set of participants in the training, validation, or test sets. Furthermore, the large number of cough samples and participants in comparison to former studies [7,24,25,30,31] may help mitigate the risk of overfitting.

Training, validation, and test sets were created in the following way. From participants with complete data sets, roughly 20% were drawn at random; these nocturnal audio recordings constituted the testing corpus for our evaluations. From the remaining participants, roughly 15% were drawn at random to be included in the validation set. The remaining participants then comprised the training set. Thus, data were roughly split into a ratio of 65:15:20. For model evaluation, the neural network was first trained on the training set. Hyperparameter tuning and model selection were performed on the validation set. Once the best performing parameters and model were selected, the final training was completed on the unified training and validation set. Model results were derived from the test set. The data split proportions were motivated by the fact that larger amounts of training data improve the performance of the classifier [47].

#### Neural Network Architecture for Cough Recognition

**Figure 1:**
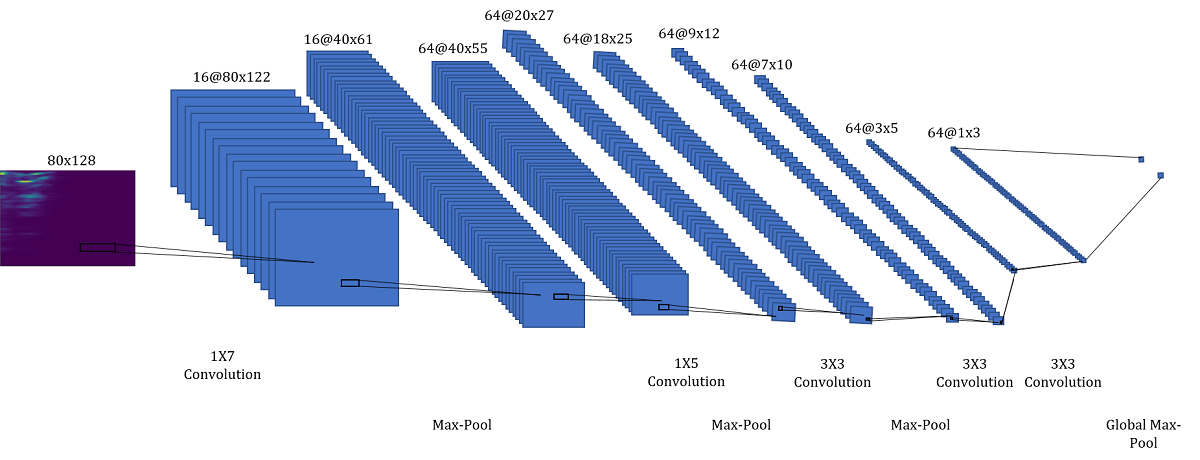
The architecture consists of 5 convolutional layers with alternating max-pooling layers followed by a global max-pooling layer. The annotation "16@80x122" refers to a feature map with dimensions (height x width) and 16 channels. The annotation "1x7 Convolution" refers to a convolutional filter with spatial dimensions (height x width).

This work was built upon a convolutional neural network architecture for cough recognition that we introduced in previous work [32] which recognized coughs in Mel spectrograms. Mel-scaled spectrograms are visual representations of audio signals with respect to time and frequency. Signal frequency ranges are Mel-scaled to represent the human perception of sound. In conjunction with convolutional neural network architectures, they have been reported to perform better than other time-frequency representations [48]. The detailed calculation of the Mel spectrograms used can be found in Multimedia Appendix 1. We evaluated this approach against different approaches for smartphone-based cough recognition in previous work on voluntary coughs and found that it performed best [32]. Our approach produced stable results across recordings of five different devices with different hardware and service life duration. Moreover, it was designed to be lightweight and its deployment and energy-efficiency were tested on smartphones. Figure 1 depicts the architecture.

#### Network Training for Cough Recognition

##### Overview

A common problem in real data sets is that some classes have more samples than others. This class imbalance can have a considerable detrimental effect on convergence during the training phase and generalization of a model on the test set [40]. To counter the high imbalance in our data set, we employed 3 different techniques: ensemble learning, balanced minibatch learning, and decision thresholding. Our training approach can be summarized as follows. First, equally sized windows were extracted from the labeled acoustic cough and noncough events. From the resulting windows, we computed Mel spectrograms and employed data partitioning on the cough and noncough Mel spectrograms. Second, we created 5 folds out of the training data. Third, from each of those folds, a separate convolutional neural network model was trained using balanced minibatch training. These models had a probability score as output that indicated the likelihood of a cough. Finally, a threshold was determined to determine the averaged predicted probability of the trained models. The trained ensemble convolutional neural network classifier including thresholding is referred to as ensemble convolutional neural network throughout this paper.

##### Window Extraction

To train the classifier, nonoverlapping 650 ms windows from the noncough acoustic events were extracted. The duration of the window was based upon previous work. The same approximate duration has performed best in other cough monitoring approaches [49]. From the cough events, a single 650 ms window centered around the maximum amplitude was extracted since most cough events were shorter. Mel spectrograms were computed for these windows.

##### Ensemble Learning

In ensemble learning, combining individual models may help to improve generalization performance, if the individual models are dissimilar [50]. In previous work [32], we employed this approach by varying the subset of the devices from which single classifiers are trained, and thus created dissimilar classifiers. In this data set, we had a vast amount of recordings from one device model at our disposal. We, therefore, aimed to create dissimilar classifiers by training disjunct folds of the data instead. At the same time, by using disjunct folds, we reduced the training duration for a single classifier. Ensemble classifiers have also been applied successfully to other imbalanced data sets in prior work [38]. In this work, we subsampled the noncough class of the training data set into 5 participant-disjunct parts. For each of these subsamples and for the total sample of coughs in the training data, we trained a separate convolutional neural network classifier. In such a manner, we reduced the level of imbalance and still benefited from the increased prediction performance of an ensemble classifier. We limited our approach to 5 classifiers in order to limit computation, which increased linearly with the number of classifiers. To execute one of the convolutional neural network classifiers, 10.74 million floating-point operations are needed. The amount of computation may be of importance if the model is to be deployed on smartphones with limited computational resources. The output of each classifier is a probability score computed in the sigmoid neuron of the architecture.

##### Balanced Minibatch Training

Although the multilayer architecture of neural networks is essential, it is the back-propagation algorithm [51]that solves the optimization problem of minimizing the error between the samples and the predictions during the training phase using gradient descent. It computes the gradients of this error with respect to the neural network weights and propagates it back over the different layers defined by the architecture.

This process was done iteratively using the learning rule defined as follows:


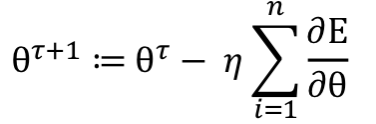


where *n* was the number of samples, *θ^τ+^*^1^ was the new updated weight, *θ^τ^* was the current weight, *η* was the learning rate, *E* was the error, and ∂ was the differential operator. A more efficient technique for the evaluation of the learning rule is minibatch training [39] which introduces a trade-off when updating the weights. While using all training samples at once would have allowed for a precise update of the weights, it would also have taken longer and would have required excessive memory, and using a single instance to update the weights would have introduced noisy updates and would have been computationally inefficient. Minibatch training, however, splits the training data into small batches and computes weight updates from those batches. We employed balanced minibatch training, which also balanced the amount of majority and minority class instances per batch. Finally, we trained each of the convolutional neural network classifiers using balanced minibatch training and the Adam adaptive learning rate optimization algorithm [52]. This training approach relied on a number of hyperparameters, which were tuned and selected on the validation set, learning rate, batch size, and the number of iterations. Furthermore, weight initialization was accomplished using Xavier initialization [53] and dropout which is a regularization technique to prevent overfitting [54]. A dropout rate of 50% was used.

##### Thresholding

Thresholding adjusts the decision threshold of a classifier. It is typically applied in the test phase and involves changing the output class probabilities [40]. Since we used balanced minibatch training, in the training phase, we implicitly assumed that cough and noncough windows were each as likely to occur as the other. To account for the class imbalance and to find the best decision threshold, we employed a grid search on probabilities in the range of [0.5, 1) and tuned it on the validation set. The single classifier is one randomly selected classifier out of the 5 classifiers that constitute the ensemble classifier. We computed the threshold-based decision rule for the ensemble classifier as follows:


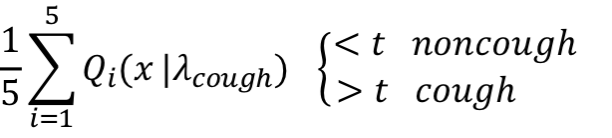


where *Q_i_*(*x*|*λ_cough_)* is the probability score of the classifier *i* for the Mel spectrogram *x* and the class cough *λ_cough_*, and *t* is the threshold.

#### Cough and Cough-Epoch Segmentation

**Figure 2:**
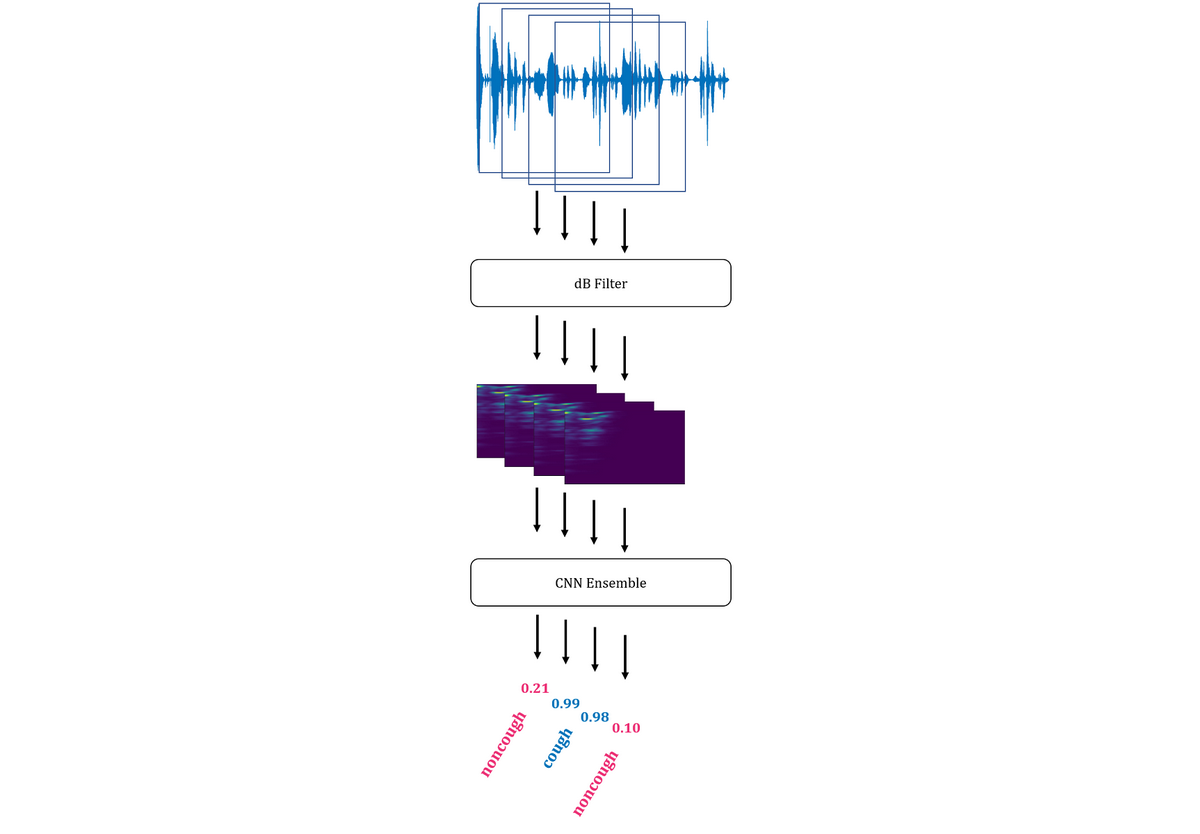
The steps for the segmentation of coughs from continuous audio recordings (from top to bottom): First, the continuous extraction of overlapping windows from continuous audio recordings; second, the discarding of silent windows by applying a dB filter; third, the computation of Mel spectrograms; fourth, the computation of the prediction probability of cough by the convolutional neural network ensemble; last, the recognition of cough by applying the postprocessing rules. CNN: convolutional neural network.

In cough segmentation, the objective is to segment coughs from continuous audio recordings by employing a trained convolutional neural network ensemble classifier. We extracted 650 ms window with an overlap of 65 ms. To discard silent windows, each of these windows passes through a decibel filter that removes windows with sounds below –26 dB as in the annotation process. Subsequently, preprocessed into Mel spectrograms for each window were computed (as described in Multimedia Appendix 1). From these Mel spectrograms, cough probabilities were computed using the trained ensemble classifier. The continuous probabilities that were output were then transformed into segmentations by applying 3 postprocessing rules: (1) Only consecutive probabilities above the derived threshold were considered to be coughs, to reduce the number of false detections. (2) Single probabilities above the threshold were then considered, when the mean of the probability above the threshold and the following probability was above 0.9. (3) When more than eight consecutive detections occurred, they were recognized as two coughs. This was done to compensate for the limited resolution caused by the size of the overlap. These rules were derived by observation and yielded the best results on the validation set. Figure 2 illustrates the cough segmentation process. From the segmentation of cough, cough counts were computed*.* Cough epochs were recognized when two (or more) coughs occurred without a 2-second pause in between. An annotated cough epoch was considered to be correctly identified when at least one of the predicted coughs of the cough epoch corresponded with the annotated cough in the cough epoch.

### Sex Classification

#### Data Set Partitioning

For the data set partitioning for determining the source of each cough by sex, we used the complete data set and did not consider the partitioning used for cough recognition. The reason for this lay in the limited amount of data that fulfilled the filtering requirements for the analysis. Since the data collection study included couples or multiple people in one room, we filtered the annotated cough data based on the information collected daily regarding whether the participant slept alone or not. We then filtered the data of the corresponding nights to create a balanced data set of male and female coughs that included 19 female and 19 male participants. We conducted our analysis on both extracted cough and cough-epoch signals. In both cases, we partitioned the data set into a disjunct training set of 10 female and 9 male and a test set of 9 female and 10 male participants.

#### Gaussian Mixture Models

Gaussian mixture models in combination with Mel-frequency cepstral coefficients [55] are a known method for tackling several different recognition tasks in the audio domain, such as text-independent speaker recognition [56] or gender recognition from speech [57]. For the sex classification of cough signals, we used the 650 ms windows that were labeled as coughs and the resulting cough epochs to compute the Mel-frequency cepstral coefficients. In addition, their first time-derivative estimate and the first time-derivative estimate of the zero-crossing rate computed over the signal were used as features. The idea of training Gaussian mixture models was to approximate the probability distribution of a class by a linear combination of *K* Gaussian distributions. The likelihood function of feature vector *X* given class *λ* can be described as follows:


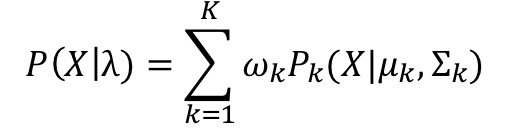


where *P_k_*(*X*|*µ_k_*, Σ*_k_)* is the Gaussian distribution. The parameters mean *µ_k_*, covariance Σ*_k_*, and weights *ω* of the distributions are determined during the training of the features *X* of class *λ*.

Considering the equal distribution of both sexes in the partitioned data set, a feature vector *x*_i_ of a cough or cough epoch can then be evaluated as follows:


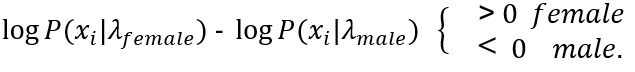


Mel-frequency cepstral coefficients (n=20) were computed with 256 samples between successive frames and a 4096-point fast Fourier transform. Analogously, the zero-crossing rate was computed over frames of 4096 samples with 256 samples between successive frames. These features were then vertically concatenated, which resulted in a matrix where the first dimension contained 41 entries. Feature selection and specific parameters were determined by employing 5-fold cross-validation on the training set. Hyperparameters of the Gaussian mixture models were also determined by employing 5-fold cross-validation on the training set which resulted in 30 Gaussian distributions each for female and male classes. Further hyperparameters were the number of initializations (n=3), number of expectation-maximization iterations (n=200), and the use of diagonal-type covariance.

### Performance Evaluation

For the evaluation of the performance of the different models, we reported several metrics such as sensitivity (true positive rate), specificity (true negative rate), accuracy, Matthews correlation coefficients, predictive positive value, negative predictive value, receiver operating characteristic curve, precision-recall curve, and Bland-Altman plot. These metrics are commonly used in machine learning and research in the context of clinical decision-support systems. For the segmentation of cough and cough epoch, we reported the number of false positives, true positives, and false negatives per night. These metrics are defined in Multimedia Appendix 1.

## Results

### Data

#### Participant Data

A total of 94 participants (female: 54/94, 57%; male: 40/94, 43%) were recruited for the study. Ages of the participants ranged from 18 to 89 years with a mean of 43 (SD 16) years. Fifteen of the 94 participants were excluded from the analysis; 2 participants withdrew, 3 participants were not involved in the study procedures for more than 5 days, and 10 participants had more than 5 nights of missed audio recordings. Some of the missed audio recordings were due to technical difficulties (such as the app crashed) while some were participant-related (such as the participant’s smartphone had been turned off).

#### Data Set Partitioning

##### Cough Recognition

Of the 79 participants whose data were included for analysis, 15 participants were initially drawn at random to be included in the test set. From the remaining 64 participants, 12 additional participants were drawn at random and included in the validation set. Data from the remaining 52 participants comprised the training set.

#### Window Extraction

Of a total of 704,697 acoustic events, 30,304 were clearly classified as coughing and 0.11% (767/704,697) were discarded. A total of 2,547,187 noncough and 30,304 cough Mel spectrograms were computed yielding a 0.015 class ratio.

#### Thresholds

Thresholds of 0.98 and 0.95 yielded best results in terms of Matthews correlation coefficient for the single and ensemble convolutional neural networks of 84% and 91% on the validation set, respectively.

#### Annotator Intraclass Correlation

Two of the annotators together accounted for 90.23% of all nights. These two annotators had an intraclass correlation of 95.79% (mean absolute error: 0.44 coughs per night). We calculated the intraclass correlation based on 65 nights. The intraclass correlation was interpreted as excellent.

### Evaluation Cough Recognition Classifier Performance

**Figure 3:**
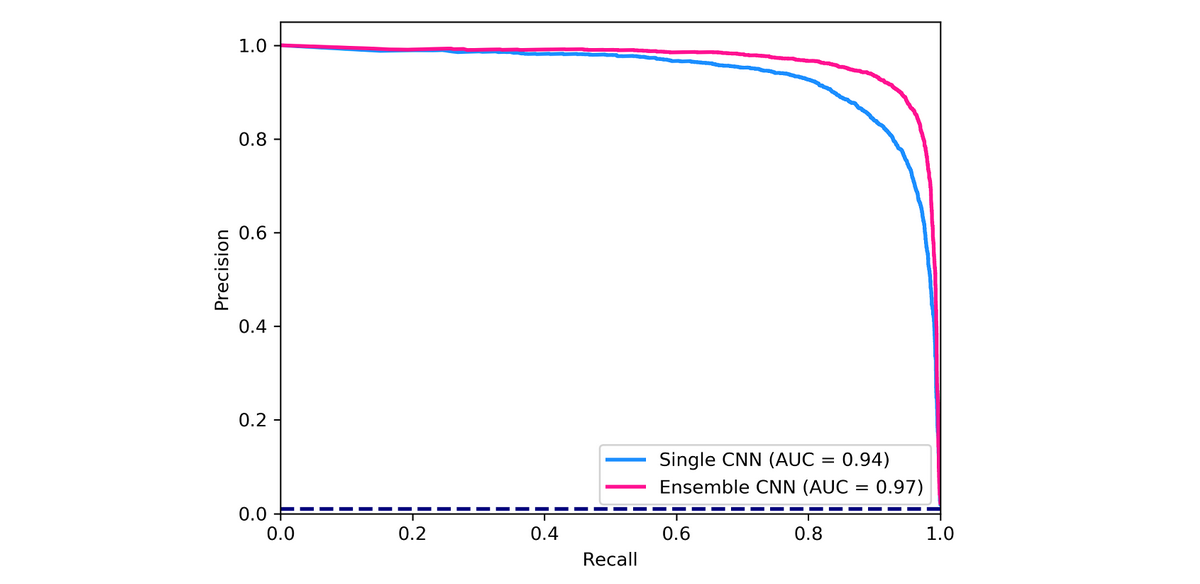
Precision-recall curves with the corresponding area-under-the-curve values, for the single and ensemble convolutional neural network models for the recognition of coughing. The dashed line represents the curve for a random classifier showing the proportion of cough-class instances to the total amount of instances. AUC: area under the curve; CNN: convolutional neural network.

We evaluated the classifiers on the testing set, which consisted of 5489 cough and 541,972 noncough events. The test set represented the soundscape encountered in the bedrooms of 15 participants over the course of 28 nights. As shown in Table 1, the performance of the ensemble classifier was better than that of the single classifier. The difference was especially notable for true negative rate, negative predictive value, Matthews correlation coefficient values, and the area under the curve of the precision-recall curve. Both classifiers showed better performance for true positive rate compared to that for true negative rate which indicated a superior capability to recognize cough whenever a cough sound was presented compared to the capability to recognize noncough sounds. As a consequence, the false positive rate was higher than the false negative rate (Figure 3).

**Table 1:** Results of the convolutional neural network classifier for cough recognition.

Model type	True positive rate, %	True negative rate, %	Accuracy, %	Matthews correlation coefficient, %	Positive predictive value, %	Negative predictive value, %
Single	99.9	87.5	99.7	87.2	99.9	87.1
Ensemble	99.9	91.5	99.8	92.0	99.9	92.6

### Evaluation Cough and Cough-Epoch Segmentation

**Figure 4:**
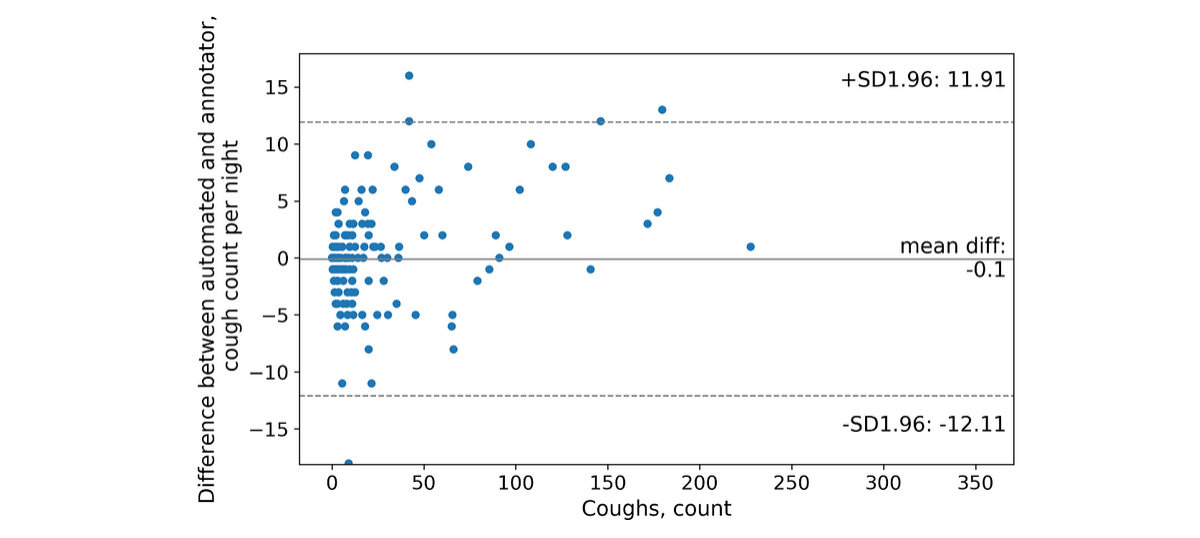
Bland-Altman plot of the automated and annotator cough counts per night.

**Figure 5:**
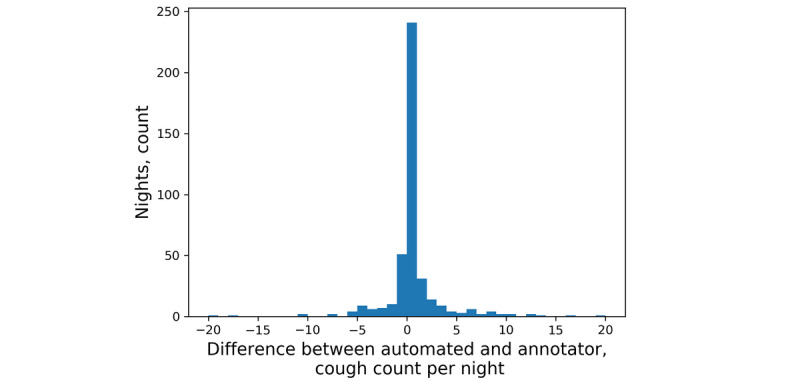
Histogram of the differences between automated and annotator cough counts per night.

**Figure 6:**
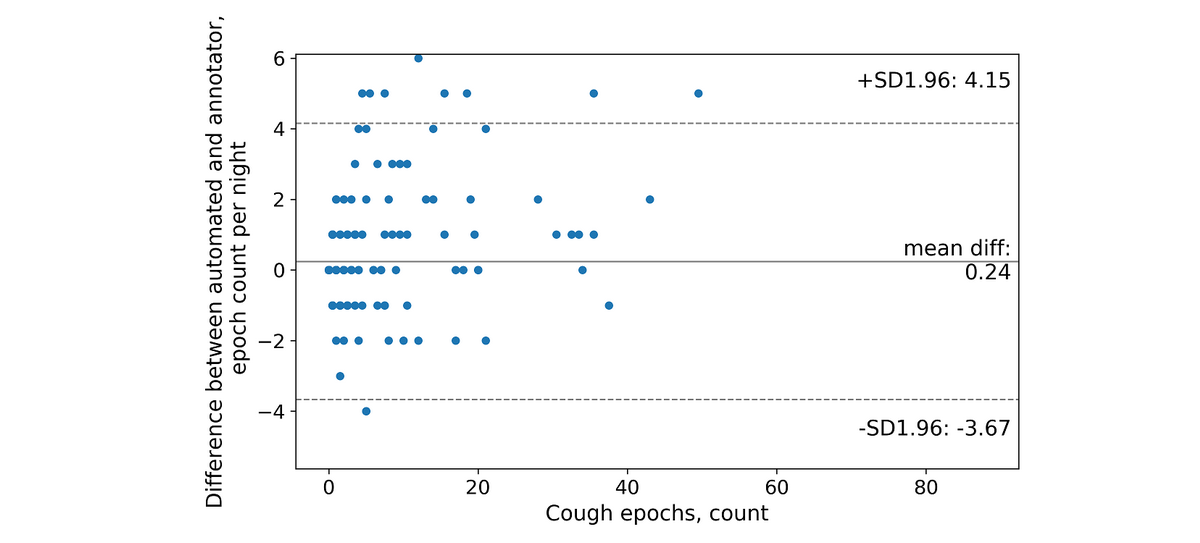
Bland-Altman plot of the automated and annotator cough-epoch counts per night.

**Figure 7:**
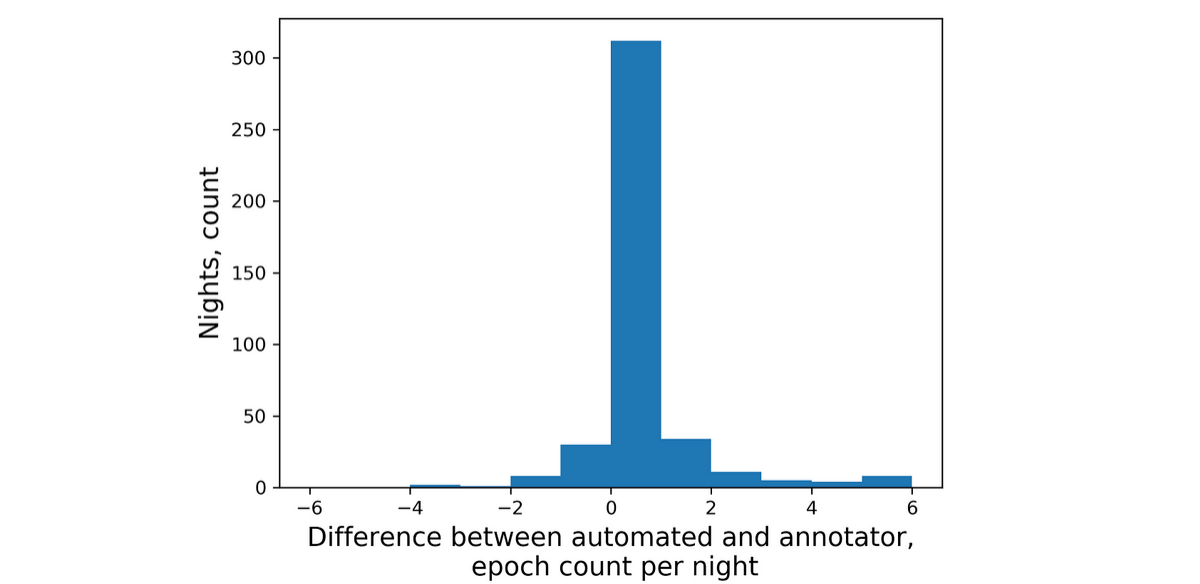
Histogram of the differences between automated and annotator cough-epoch counts per night.

The test set included 15 participants and 421 nights; human annotators counted as few as zero and as many as 368 coughs in one night. The mean count difference between automated and annotator coughs was –0.1 (95% CI –12.11, 11.91) coughs per night (Figure 4). Further, our classifier produced a mean of 1.76 false positives per night and 1.66 false negatives per night. In total, 241 nights were identified with a count difference of 0 coughs (Figure 5) and 5 nights had count differences greater than 20 coughs. Human annotators counted as few as zero and as many as 101 cough epochs in one night. The mean count difference between automated and observer cough epochs was 0.24 (95% CI –3.67, 4.15) epochs per night (Figure 6). Our classifier produced a mean of 0.33 false positives per night and 0.57 false negatives per night. In total, 312 nights were identified with a count difference of 0 cough epochs (Figure 7) and 6 nights had count differences greater than 6 cough epochs.

### Evaluation of Sex Classifier Performance

Using the data set that included 19 female and 19 male participants which had been selected for a balanced set of male and female coughs, both extracted cough and cough-epoch signals were analyzed. The partitioning resulted in 1532 female and 1527 male coughs for training and 500 female and 498 male coughs for testing. In the case of cough epochs, this partitioning led to 366 female and 351 male cough epochs for training, and 194 male and 134 female cough epochs for testing.

**Figure 8:**
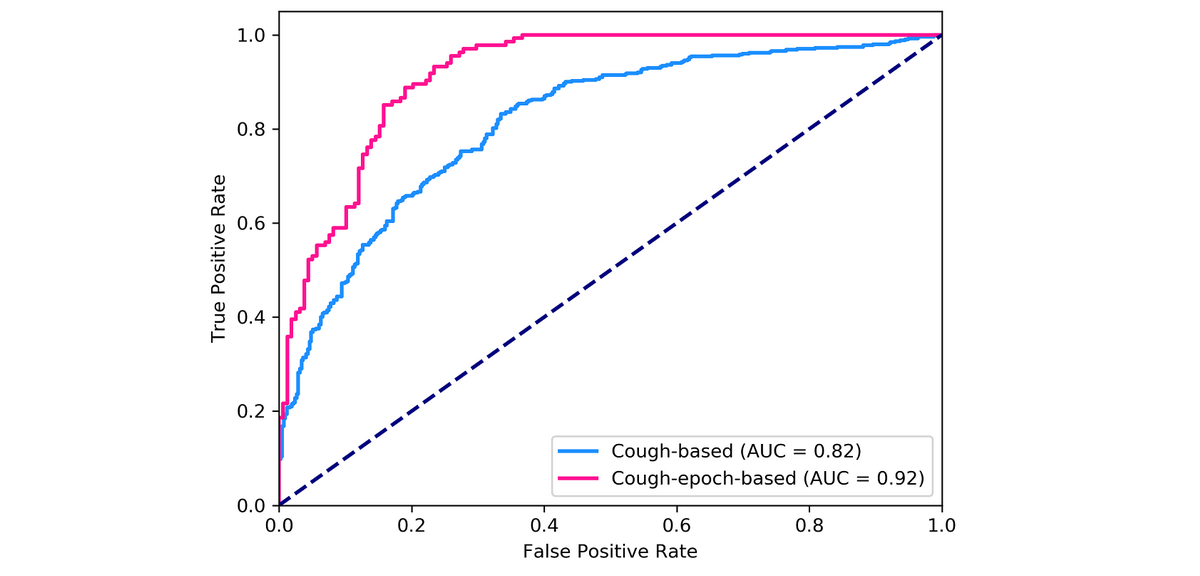
Receiver operating characteristic curves with corresponding area-under-the-curve values for cough and cough epoch–based sex assignment. The dashed line represents the curve for a random classifier. AUC: area under the curve; ROC: receiver operating characteristic.

As shown in Table 2 and Figure 8, the performance of the classifier that was based on cough-epoch signals outperformed the classifier based on cough signals. The difference was especially notable on true positive rate, accuracy, Matthews correlation coefficient, positive predictive values, negative predictive values, and the area under the curve of the receiver operating characteristic curve. Both classifiers showed better performance for true positive rate than for that of true negative rate, which indicated a superior capability to recognize female cough or cough-epoch signals whenever a female cough or cough-epoch signal presented itself, in comparison to the corresponding capability to recognize a male cough or cough-epoch signal.

**Table 2:** Gaussian mixture model results of sex recognition for coughs and cough epochs.

Model for	True positive rate, %	True negative rate, %	Accuracy, %	Matthews correlation coefficient, %	Positive predictive value, %	Negative predictive value, %
Cough	81.0	71.8	74.8	49.6	57.8	88.8
Cough epochs	95.0	74.9	83.2	69.1	72.8	95.5

## Discussion

### Principal Findings

To the best knowledge of the authors, the data set in this paper is the largest, real-life cough data set with published recognition and segmentation results, not only for adults with asthma but across all respiratory conditions. Given the data set of continuous overnight recordings of 79 adults with asthma in different soundscapes (excluding dropouts), our results demonstrate that cough recognition from smartphone-based audio recordings is feasible. The ensemble classifier performed well with values greater than 90% across different metrics for the pure classification task and achieved comparable cough counts to that of human annotators in the segmentation of coughing from continuous overnight audio recordings. In specific cases (for example, the 6 nights with a difference in cough counts of 20 and above), a need for further development was demonstrated. We listened to the original recording of these cases and believe these failures were caused by strong background noise, peculiar chuckle and laughter sounds, and a specific type of music, among others. These sounds, however, strongly suggest that the participant was not asleep.

We also provided a first step towards distinguishing partner cough from patient cough by determining the source of cough signals classifying those that corresponded to sex of the patient as patient coughs. This can be applied to cough recordings from the bedrooms of opposite-sex couples, even when both are coughing. Our results further indicate that cough epoch–based sex classification has greater potential than that of cough-based sex classification. This may be explained by the fact that cough epochs are longer and may contain more periodic information, rather than the limited amount of periodic information contained in the short bursts of the explosive cough sound. Speech signals of a typical adult male contain a fundamental frequency from 85 to 180 Hz and those of a typical adult female from 165 to 255 Hz [58]. This discrepancy gives rise to sex classification from speech with greater than 90% of accuracy [59]. Finally, we also investigated the automatic segmentation of cough epochs from continuous audio recordings which yielded results that were comparable to those of annotated cough epochs. These results and the fact that 86% of the coughs in our study originated from cough epochs provided a foundation for cough epoch–based sex classification. Our classifier determined the source of cough epochs based on sex with 83% of accuracy.

### Comparison With Prior Work

Cough-monitoring systems that are capable of detecting reflex coughs in audio recordings have been proposed in previous work [24,25,30,60], and some of them have achieved sensitivity (true positive rate) and specificity (true negative rate) values greater than 90% [25,30,60]; however, these data sets contain coughs that have been recorded in various conditions and that have been applied in different contexts which makes a comparison with our work difficult. To our knowledge, no models or systems have been trained and evaluated on such an extensive asthmatic cough database; our data set contained 30,304 coughs. None of these systems underwent longitudinal evaluation (for more than one night). For instance, the Leicester Cough Monitor system was one of the few systems that was evaluated over a longer period of time; 6-hour and 24-hour cross-sectional recordings of patients with chronic cough [25]. Only a few approaches proposed modes of use that were comparable to our mode of use where the microphone was not attached to the patient [24,31,60]. Among those, only one involved a smartphone, where 1-hour recordings were collected for each participant in a laboratory setting [31,61]. None of these approaches addressed the problem of distinguishing the participant’s cough from the coughs of other people in contact-free recordings.

### Limitations

There were several limitations in our study regarding the generalization of our results. First, we only used data collected by one specific model of smartphone. It has previously been demonstrated [32] that noisy or low-quality recordings from a different device can have a detrimental effect on the performance of the classifiers. Second, the data set may limit the generalizability of our sex-classification results. For the analysis, we included data from different male and female participants, who slept alone but were recorded in different rooms, in contrast to a real scenario of a couple, where both are sleeping and coughing in the same room. Due to the amount and length of the recordings, the annotation process was extremely laborious. As a consequence, the majority of the recordings were only annotated by one annotator. This gives rise to the possibility that certain coughs were missed or wrongly annotated.

### Conclusions

Our study proposed a combined approach to combat the detrimental effect of learning from highly imbalanced data sets by combining techniques such as ensemble learning, balanced minibatch training, and decision thresholding. We showed that automated methods can recognize nocturnal coughs and cough epochs in smartphone-based audio recordings. The model addressed distinguishing subject coughs from those of a bed partner in contact-free recordings by classifying cough and cough-epoch signals to the corresponding sex of the participant. This research enables smartphone-based cough monitoring of individuals and of couples of different sexes in their bedrooms. It represents a step towards passive, scalable cough monitoring for people with asthma, and thus contributes to the development of a scalable diagnostic tool for early recognition of exacerbations.

## Appendix

Multimedia Appendix 1 Additional information.
