# Supplementary material for: Automatic Recognition, Segmentation, and Sex Assignment of Nocturnal Asthmatic Coughs and Cough Epochs in Smartphone Audio Recordings: Observational Field Study
Source: J Med Internet Res. 2020 Jul 14;22(7):e18082. doi: 10.2196/18082 (PMC7388043; doi:10.2196/18082)
Supplement: Multimedia Appendix 1 [file jmir_v22i7e18082_app1.docx]

## Multimedia Appendix 1

### Appendix I: Recording Settings

Nocturnal smartphone audio data was recorded by default from 23:00 hours for a recording duration of 9 hours and 40 minutes to take into account the sleep patterns of the general Swiss population [62] (i.e., 95% of nightly sleep sessions are shorter than this standard recording duration). Recording occurred passively in the background without any interaction of the participant. However, participants could manually start (after 9 p.m.) and stop (after 4 a.m.) audio data recording, overwriting the default recording settings. If a participant started recording manually after recording had already started automatically (e.g., after 23:00), the default recording time would be extended to 9 hours and 40 minutes from the time of manual initiation. The data recorded between 23:00 and the point of the manual start was not considered for the analysis. Also, the app was designed to record all the sounds at 16 kHz and 16 bits/sample, respectively.

### Appendix II: Annotation

A total of four annotators labeled the whole dataset. Annotators one, two, three, and four labeled 69%, 22%, 7%, and 2% of the recorded audio data, respectively. Also, annotator one finalized uncompleted or missing annotations. We used the label of the annotator to first label the data as ground truth for recordings with multiple annotations for the computation of the ICC. Finally, all annotators were equipped with a labeling instruction manual. We have included the original and a translation of the instruction manual as supplementary documents. We explained the manual in the first session of the annotation process, answered questions, and performed demonstrations.

### Appendix III: Data Preparation and Preprocessing for Cough Recognition

We followed the preprocessing steps that we have introduced and optimized in previous work [32]. The extracted windows of any sound event, cough and non-cough, were multiplied by a Hanning window, subsequently filtered by a Butterworth high-pass filter of order 5 and a cut-off frequency of 10 Hz to reduce lower-band noise and discontinuity effects. The filtered signal was then standardized by employing min-max normalization. Finally, Mel-scaled spectrograms were computed with 80 bands, 112 samples between successive frames and a 2048 point FFT yielding an 80x128 sized matrix.

### Appendix IV: Evaluation Metrics

#### True Positive Rate (TPR), also known as ‘Recall’ or ‘Sensitivity’

It is the number of true positives (TP) divided by the sum of true positives (TP) and false negatives (FN) [63]:

$$TPR=\frac{TP}{TP+FN}$$

In the case of cough recognition, TPR refers to the percentage of the correctly predicted coughs to the total number of coughs in the test set.

#### True Negative Rate (TNR), also known as ‘Specificity’

It is the number of true negatives (TN) divided by the sum of true negatives (TN) and false positives (FP) [63]:

$$TNR=\frac{TN}{TN+FP}$$

In the case of cough recognition, TNR refers to the percentage of the correctly predicted non-cough sounds to the total number of non-cough sounds in the test set.

#### Accuracy (ACC)

It is computed as the sum of true positives (TP) and true negatives (TN) divided by the number of all instances (N) in the dataset [63]:

$$ACC=\frac{TP+TN}{TP+FN+FP+FN}$$

In the case of cough recognition, ACC referred to the proportion of correct predictions, both cough and non-cough.

#### Matthews Correlation Coefficient (MCC)

It is a balanced metric, considering both classes as important, even if one class is disproportionally represented. It is computed by taking into account all four values in the confusion matrix, i.e., true positives (TN), false positives (FP), true negatives (TN), and false negatives (FN), as follows:

$$MCC=\frac{TP\cdot TN-FP\cdot FN}{\sqrt{(TP+FP)(TP+FN)(TN+FP)(TN+FN)}}.$$

#### Positive Predictive Value (PPV), also known as ‘Precision’

It is the number of true positives (TP) divided by the sum of true positives (TP) and false positives (FP):

$$PPV=\frac{TP}{TP+FP}$$

In the case of cough recognition, PPV referred to the percentage of the correctly predicted coughs to the total number of predicted coughs in the test set.

#### Negative Predictive Value (NPV)

It is the number of true negatives (TN) divided by the sum of true negatives (TN) and false negatives (FN) [64]:

$$NPV=\frac{TN}{TN+FN}$$

In the case of cough recognition, NPV referred to the percentage of the correctly predicted non-cough sounds to the total number of predicted non-cough sounds in the test set.

#### Receiver Operating Characteristic Curve (ROC)

The ROC is created by plotting the true positive rate (TPR) against the false positive rate (FPR) at various probability thresholds [65]. The false positive rate is calculated as FPR = (1 - TNR).

#### Precision-Recall Curve (PRC)

The PRC is created by plotting the PPV against the TPR at various probability thresholds. It is considered more informative than the ROC when evaluating binary classifiers on imbalanced datasets [66].

#### Bland-Altman Plot

It is used to compare a new measurement technique with the gold standard [67], i.e., automated cough counts with observer cough counts. In our case, each night is represented by the mean of the two measurements as the x-value and the difference between the two values as the y-value.

## Multimedia Appendix References

32. Barata F, Kipfer K, Weber M, Tinschert P, Fleisch E, Kowatsch T. Towards Device-Agnostic Mobile Cough Detection with Convolutional Neural Networks. 7th IEEE International Conference on Healthcare Informatics (ICHI 2019). 2019:1-11.

62. Walch OJ, Cochran A, Forger DB. A global quantification of “normal” sleep schedules using smartphone data. *Science advances*. 2016;2(5):e1501705.

63. Olson DL, Delen D. *Advanced data mining techniques*: Springer Science & Business Media; 2008.

64. Fletcher GS. Clinical epidemiology: the essentials: Lippincott Williams & Wilkins; 2019.

65. Fawcett T. An introduction to ROC analysis. *Pattern recognition letters*. 2006;27(8):861-74.

66. Saito T, Rehmsmeier M. The precision-recall plot is more informative than the ROC plot when evaluating binary classifiers on imbalanced datasets. *PloS one*. 2015;10(3):e0118432.

67. Bland JM, Altman DG. Measuring agreement in method comparison studies. *Statistical methods in medical research*. 1999;8(2):135-60.
